# Peer Review of “A Local Community-Based Social Network for Mental Health and Well-being (Quokka): Exploratory Feasibility Study”

**DOI:** 10.2196/33931

**Published:** 2021-10-27

**Authors:** Hamed Mehdizadeh

**Affiliations:** 1 Department of Health Information Technology School of Allied of Medical Sciences Mazandaran University of Medical Sciences Mazandaran Iran

**Keywords:** local social network, community health, well-being, digital health, consumer health

*This is a peer-review report submitted for the paper “A Local Community-Based Social Network for Mental Health and Well-being (Quokka): Exploratory Feasibility Study.”*

## Round 1 Review

### General Comments

This article [1] is about the feasibility of using a social network to change behavior in people. This study is interesting and significant in terms of the subject and the work it does, but this article does not have the usual scientific article structure.

### Specific Comments

#### Major Comments

1. In the *Methods* section, the authors do not provide any information about how to conduct the study. Important information such as the technical information of the system or application and modules related to the application Quokka are not mentioned.

2. There is no information about how the application works. Also, the method of conducting research is only mentioned in general in the *Abstract*, and in the body of the article, no information is provided in this section.

3. In the *Methods* section, there is no clear information about how to select the statistical population, inclusion and exclusion criteria, study start and end times, etc.

4. The *Methods* section is unstructured and contains too much detail and irrelevant information about the study, which not only does not help the audience to understand more but also confuses the reader.

5. Therefore, it is recommended that the *Methods* section be completely rewritten and structured like other scientific articles.

6. In the *Results* section, although the results are well described in the table, some of this information seems to be added.

7. In the *Discussion* section, there is no information about similar studies.

8. The results obtained in this study have not been compared with previous or similar studies, and the strengths and weaknesses of the study have not been expressed.

## Round 2 Review

First of all, the authors did not answer of any of my comments.

I suggest that the authors read similar papers published in JMIR to better understand how to report their study like a scientific report and write their manuscript based on these papers.

In the *Introduction*, the semantic structure and hierarchy that are needed for a scientific report do not exist.

The *Introduction* should include the problems, existing solutions, and choice of solutions based on logical reasons and references to studies that use it.

In this section, the authors should explain what issue needs to be solved and what can be done to solve this issue (method or tools or...) as well as describe why they chose the selected method for this problem or issue. Then, they should refer to similar studies that have used these methods. In fact, the introduction should answer these questions: What is the problem? What are the ways to solve it? Talk about your choice and the logic behind that.

In the second paragraph, the author says:

“Here, we discuss Quokka's challenge, local...”

This information has nothing to do with this section and should be transferred to the *Results* or *Discussion* section.

The first part of the *Introduction* before *Related Work* only contains one scientific reference, and the rest of the contents are without references. In an introduction, you can’t say anything without references.

In the *Introduction*, paragraphs 2, 3, and 4 are not related to this section and must be removed.

At the end of the first paragraph, the author refers to health behavior change; it is necessary to refer to the role of behavior changing theory (BCT) and its application in health interventions. Also, refer to similar studies that have used this approach to change health behaviors.

At the end of paragraph, the author says:

“in contrast with prior works”

Which prior works? You do not say anything about other studies in this field, weaknesses, gaps, or anything else.

In the last paragraph of the *Introduction* or after this section, the aim of the study should be mentioned.

None of the comments that I have mentioned in the previous review have been resolved. My point is that the *Methods* section requires rewriting. This time, I mention the items that must be corrected in more detail, and I hope they will be fixed by the authors.

In the first part of the *Methods*, the information provided has nothing to do with this section.

For example, all content from first paragraph in the *Methods* to the end of *Recruitment* are irrelevant.

In the *Methods*, you should provide information about how you designed the study, like this:

How to design the study?

How do you want to do that?

What is the sampling method?

What were the inclusion/exclusion criteria?

Have people signed consent forms to participate in study?

How was information collected, and what method or instrument has been used?

What is the time period of this study? etc.

In the *Methods*, the author says that the purpose of this program is to change healthy habits, in other words, change behaviors of people.

However, there is no information about the mechanism of behavior change or use of behavior change theories in their intervention.

This is very important in that the intervention aimed to change behavior using behavioral change theories and techniques.

Also, when your main purpose of the design of this program is to change people’s behaviors, you should provide information about these techniques and the efficiency of these methods to solve the problem you speak about.

The paper has no *Results* section. The *Methods* section and the results are combined.

In the *Discussion*, the results are interpreted incompletely and are not compared with the results of other studies.

*Limitations and Weaknesses* is unstructured and too long, with unrelated content. Generally, in the “limitations,” the author discusses the weaknesses of the study, including the low number of samples, conducting the study in one center, etc.

The references list, generally, must be rewritten.

I suggest looking at the references list in similar papers published in JMIR so that you can better understand the structure of a scientific article and try to submit your report based on the overall structure of these articles.

I suggest looking at the references list in similar papers published in JMIR and rewrite this section based on the journal format requested.

Reference No. 1, the authors’ names must be modified.

Reference No. 2 should also be modified like Reference No 1.

Reference No. 7, after the name of the journal, enter the publication year, not the issue or page number. This problem is seen in most cases, for example:

7. HUNT, JUSTIN, AND DANIEL EISENBERG. “Mental Health Problems and Help-Seeking Behavior Among College Students.” Journal of Adolescent Health 46, NO. 1 (2010): 3-10.

Do not write the authors' full name and last name together, for example, in References No. 10, 11, and 13.

If the authors of the study number more than 6 people, you must use “et all.” after the name of the sixth author.

## Review Round 3:

### General Comments

The revised version addresses the earlier comments and represents an improvement over the prior version.

However, in the References section, there is a minor comment.

### Minor Comments

I suggest to the authors to look at the references list in similar papers published in JMIR and rewrite this section based on the journal format requested, like this:

Reference No. 1:

Lake J, Turner MS. Urgent Need for Improved Mental Health Care and a More Collaborative Model of Care. Perm J. 2017;21:17-024. doi: 10.7812/TPP/17-024. PMID: 28898197; PMCID: PMC5593510.

Please rewrite all references like this.

## Abbreviations

BCT: behavior changing theory
